# TLQP-21 modulate inflammation and fibrosis in a model of ards

**DOI:** 10.1186/2197-425X-3-S1-A562

**Published:** 2015-10-01

**Authors:** F Pozzi, L Rizzi, V Zambelli, L Molteni, M Cavagna, P Verdie, J-A Fehrentz, J Martinez, A Torsello, G Bellani, A Pesenti

**Affiliations:** Department of Health Science, Università degli Studi di Milano Bicocca, Monza, Italy; Institut des Biomelecules Max Mousseron, Montpellier, France

## Introduction

TLQP-21 is a neuropeptide expressed in the brain that is involved in the control of energy homeostasis. In preliminary experiments we have observed that TLQP-21 can modulate macrophage function. In Acute Respiratory Distress Syndrome (ARDS) macrophage seems to play a critical role, contributing to lung remodeling.

## Objectives

To explore the therapeutic role of a short analog of TLQP-21 (JMV5656) in an experimental model of ARDS.

## Methods

C57/BL6 mice received an instillation of 0.1 M HCl, 2.5 ml/kg into the right bronchus. They were treated with TLQP-21 0.6 mg/kg ip or vehicle control, 2 days before and on the same day of HCl challenge. Respiratory system compliance, blood gas analysis and differential cell counts in a selective bronchoalveolar lavage (BAL) were determined 24 h after HCl. In a parallel experiment mice were observed for 14 days to assess epithelial damage and lung fibrosis.

## Results

**Figure 1:**
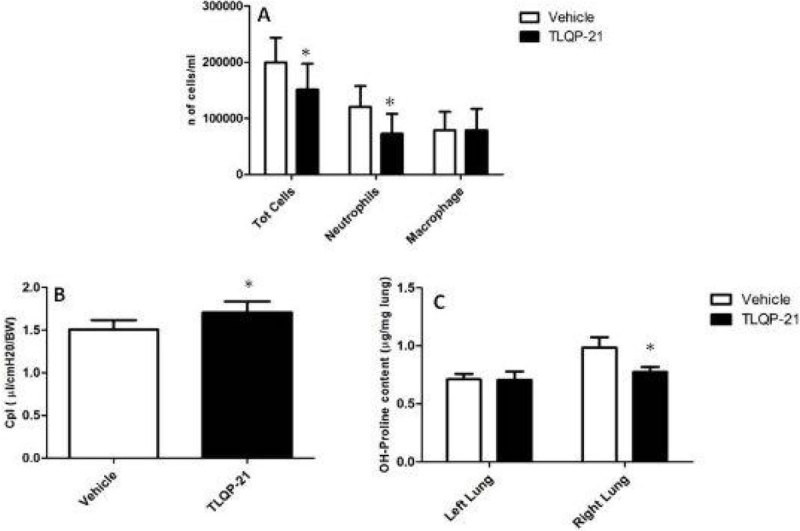
**TLQP-21 module lung inflammation and fibrosis**. A) Lower neutrophil count in the BALF of the right bronchus at 24 hours after HCI. B) At day 14 lung compliance improved. C) Collagen deposition was lower in the mice treated with TLQP-21. * p ≤ 0.05.

The treatment with TLQP-21 showed a significant decrease in the number of total cells in BALF, due to a lower recruitment of neutrophils at 24 hour after challenge with HCl, compared to the vehicle group (Figure 1A), with no differences in macrophage number , even if this did not translate in a functional improvement in lung compliance and oxygenation. At day 14 the TLQP-21 group showed an improvement in lung compliance (Figure 1B) and a decrease collagen deposition in lung tissue (Figure 1C).

## Conclusion

TLQP-21 can decrease inflammatory response at an early phase in a mouse model of HCl-induced ARDS, which may modulate lung remodeling at a late phase, preventing a fibrotic evolution. Given these encouraging but not definitive results we aim to furtherassess the potential therapeutic effect of a higher dose of TLQP-21

